# Imputation methods for missing outcome data in meta-analysis of clinical trials

**DOI:** 10.1177/1740774508091600

**Published:** 2008

**Authors:** Julian PT Higgins, Ian R White, Angela M Wood

**Affiliations:** MRC Biostatistics Unit, Institute of Public Health, Robinson Way, Cambridge, UK

## Abstract

**Background:**

Missing outcome data from randomized trials lead to greater uncertainty and possible bias in estimating the effect of an experimental treatment. An intention-to-treat analysis should take account of all randomized participants even if they have missing observations.

**Purpose:**

To review and develop imputation methods for missing outcome data in meta-analysis of clinical trials with binary outcomes.

**Methods:**

We review some common strategies, such as simple imputation of positive or negative outcomes, and develop a general approach involving ‘informative missingness odds ratios’ (IMORs). We describe several choices for weighting studies in the meta-analysis, and illustrate methods using a meta-analysis of trials of haloperidol for schizophrenia.

**Results:**

IMORs describe the relationship between the unknown risk among missing participants and the known risk among observed participants. They are allowed to differ between treatment groups and across trials. Application of IMORs and other methods to the haloperidol trials reveals the overall conclusion to be robust to different assumptions about the missing data.

**Limitations:**

The methods are based on summary data from each trial (number of observed positive outcomes, number of observed negative outcomes and number of missing outcomes) for each intervention group. This limits the options for analysis, and greater flexibility would be available with individual participant data.

**Conclusions:**

We propose that available reasons for missingness be used to determine appropriate IMORs. We also recommend a strategy for undertaking sensitivity analyses, in which the IMORs are varied over plausible ranges.

## Background

Clinical and policy decisions regarding healthcare interventions are increasingly based on evidence from meta-analyses of randomized controlled trials (RCTs). Threats to validity of RCTs carry through to meta-analyses containing them. Well known threats include poor concealment of allocation and inappropriate blinding of participants and personnel [1]. A further threat that has received relatively little attention in the meta-analysis context is missing outcome data. RCTs almost inevitably fail to collect relevant outcome data on every randomized participant, no matter how rigorous their methodology. Missing outcome data lead to increased uncertainty over the effect of an intervention, and if ignored may lead to biased estimates. A full intention-to-treat (ITT) analysis is often interpreted as including all randomized participants, and such analyses should recognize and incorporate the implications of missing observations [2].

Methodology for dealing with missing outcome data is well developed for individual RCTs, although perhaps seldom applied, with possible approaches including multiple imputation, maximum likelihood techniques and sensitivity analysis [3,4]. Much of this advanced methodology, however, requires detailed data for each participant. For example, multiple imputation and full likelihood analysis of available data are only valid if all the predictors of dropout are observed and modeled, which becomes more plausible as the data become richer.

Here we address the meta-analysis of summary data from each RCT, typically obtained from published reports. We consider simple methods in which a meta-analytic estimate is obtained as a weighted average of effect estimates [5]. Dealing with missing outcome data in a meta-analysis raises particular problems, principally arising from the limited information typically available in published reports. Although a meta-analyst would ideally seek any important but unreported data from the original trialists, this approach is not always successful and it is uncommon to have access to more than group-level summary data at best.

Meta-analyses should be replicable and therefore transparent in the methods used to derive their results, and a systematic approach to deal with missing data is desirable. In this article we focus on meta-analyses of two-arm, parallel group trials with a binary outcome. We overview methods for dealing with missing binary outcome data in clinical trials, develop and discuss them in the context of meta-analysis, and apply them to a systematic review of placebo-controlled trials of haloperidol in the treatment of schizophrenia. Our ultimate aim is to provide suggestions on the selection of a strategy, or strategies, for (i) a *primary* meta-analysis in the presence of missing data; and (ii) *sensitivity analyses* to assess the potential impact of missing data on the results.

## Methods for dealing with missing outcome data

**Figure 1:**
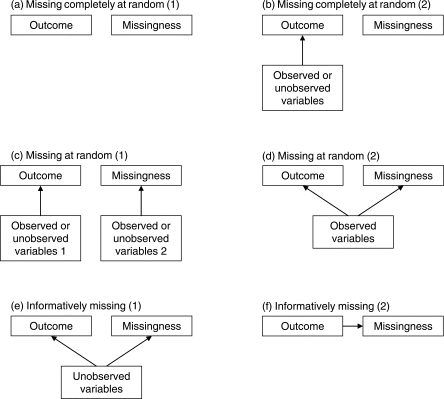
Some possible scenarios for missing data. Arrows indicate causal effects. Missing completely at random: (a) outcome and missingness are unrelated and not dependent on any other variables; (b) missingness is ‘random’, but outcome may be dependent on other variables. Missing at random: (c) different variables are responsible for outcomes and for missingness; (d) the same variables are responsible for outcomes and for missingness, but can be incorporated into the analysis; Informatively missing: (e) the same variables are responsible for outcomes and for missingness, but cannot be incorporated into the analysis; (f) missingness depends directly on the unobserved outcome

It is useful to classify missing outcome data according to the relationship between nonavailability of a particular value and the observed and unobserved values. We will use the term ‘missingness’ for the nonavailability of a participant's outcome. First, if missingness of an outcome is not related to any observed or unobserved variables, then the missing data are described as ‘missing completely at random’ (Figure 1(a) and (b)). Analysis restricted to individuals with complete data is always valid when the data are missing completely at random. If missingness of an outcome may be related to observed or unobserved variables, but is not related to the actual value of the outcome, conditional on the observed variables, then the missing data are described as ‘missing at random’ (Figure 1(c) and (d)). An alternative term is ‘ignorable’, because a correct likelihood-based analysis of all the observed data is valid [3]. (Strictly, a further condition is required, but this is true in almost all practical applications.) ‘Missing completely at random’ is a special case of ‘missing at random’. Finally, if missingness of an outcome is related to the value of that outcome, even conditional on other observed variables, then the missing data are described as ‘informatively missing’. This could be because of some common unobserved cause of both missingness and the outcomes (Figure 1(e)) or because the outcome directly causes missingness (Figure 1(f )). Alternative terms are ‘missing not at random’, ‘not missing at random’ or ‘nonignorable’, the last so called because a likelihood-based analysis of the observed data alone is typically biased [3].

With data that are informatively missing, the missing at random assumption is false by definition, but there may be other more plausible assumptions that make analysis possible. For example, with repeated measures data, the observed data may be assumed to vary around an underlying person-specific trend, and analysis can be based on the assumption that the risk of dropout depends on the underlying trend [6]. If no such assumption can reasonably be made then the data analyst has little option but to consider a model containing an unknown and unidentified parameter (for example, the difference between the mean outcome in the unobserved data and the mean outcome in the observed data) and consider a range of possible values for the unknown parameter in a sensitivity analysis [7]. From a Bayesian perspective this approach can be refined by assigning a prior distribution to the unknown parameter, giving inferences that appropriately reflect uncertainty about the missing data [8,9].

In a simple RCT with a binary outcome, treatment assignment is an observed variable that may affect outcome. If treatment assignment also affects missingness, as in Figure 1(d), then the data are missing at random. It could be that some other baseline characteristics are related both to the outcome and to the risk of data being missing. In this case the data could be missing at random (as in Figure 1(d)), so that an analysis of available outcome data, provided that it adjusted for the baseline characteristics, would be valid. However, in a meta-analysis situation, such baseline data are seldom available (as in Figure 1(e)). The basic data set from each trial in a meta-analysis situation comprises a 3 × 2 table providing numbers of participants with observed positive outcome, with observed negative outcome, or having a missing outcome, in each group (Table 1). Leaving aside the observed variable representing treatment allocation, the six situations summarized in Figure 1 reduce to two. Either there is no association between missingness and outcome (Figure 1(a)–(c)) or there is association between missingness and outcome (Figure 1(d)–(f)). In practice, the alternative options within each treatment group are therefore to treat the missing data as missing completely at random or to treat the data as informatively missing.

**Table 1:** Basic data and statistics from a single trial

	Data				Statistics	
	Event	No event	Missing	Total	Observed risk	Proportion missing
Experimental	r_E_	f_E_	m_E_	N_E_	*p*_E_ = r_E_/(r_E_ + f_E_)	a_E_ = m_E_/N_E_
Control	r_C_	f_C_	m_C_	N_C_	*p*_C_ = r_C_/(r_C_ + f_C_)	a_C_ = m_C_/N_C_

It is not possible to determine which of these approaches is more appropriate from the 3 × 2 table from a single RCT. In a meta-analysis of several RCTs with different proportions of missing data, a plot of effect estimates against the proportion of missing data, and an associated meta-regression analysis, may reveal a relationship that indicates informative missingness. This would rest on an assumption that the effect sizes underlying the complete (observed and unobserved) data are similar across trials, so that systematic deviation from this underlying effect size is due to omission of data from participants in whom the effect size is larger or smaller. However, meta-regression is an unreliable technique, particularly because of confounding [10]. For example, the more pragmatic trials in a data set may be larger, simpler and have wider eligibility criteria resulting both in higher rates of missing data and in smaller treatment effects, so that a relationship between missing data rate and effect estimate would be confounded with these other characteristics associated with trial size. Furthermore, meta-regression will typically have low power to detect any such relationship as statistically significant and has a high false-positive error rate [11].

A more realistic approach is to tackle missing data on a trial-by-trial basis, making sensible and consistent decisions about whether data are informatively missing, and if so what the missing outcomes might have been, and assessing the sensitivity of results to these decisions. In the following discussion we describe a number of possible ways to handle missing data. We assume that the effect measure used to compare the groups is either the odds ratio (OR), the risk ratio (RR) or the risk difference (RD).

### Available case analysis (ACA)

Before considering strategies for addressing missing data we mention the option of an available case analysis, which includes only those participants whose outcome data are known. This is often termed complete case analysis, and usually provides a sensible starting point. It is probably the most common in meta-analyses in practice. As we remark above, this analysis may be biased if the fact that the data are missing is related to the unobserved clinical outcome.

### Imputed case analysis (ICA)

In an imputed case analysis, missing values are filled in using specific assumptions about what might have happened to the participants. ICA yields unbiased estimates if these assumptions are reasonable, at least on average. However, care must be taken not to underestimate standard errors [4] by ignoring uncertainty about the imputed values. Possible methods are multiple imputation [12] and using specially calculated standard error formulae [13].

Here we outline several specific approaches to imputing missing outcomes in the two treatment groups, and describe an approach that makes use of available reasons for missingness. We then provide a framework that unifies all the imputation methods. The methods are summarized in Table 2.

**Table 2:** Summary of imputation strategies, with connection with IMORs. 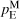 and 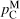 are imputed risks among missing participants based on available reasons for missingness; see text for precise definition

Method	Imputation	IMOR_E_	IMOR_C_
ICA-0	Impute missing = no event (0)	0	0
ICA-1	Impute missing = event (1)	∞	∞
ICA-p_C_	Impute all according to observed control group risk, *p*_C_	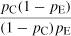	1
ICA-p_E_	Impute all according to observed experimental group risk, *p*_E_	1	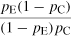
ICA-p	Impute according to observed group-specific risk	1	1
ICA-b	Impute to create best case scenario for experimental treatment	0 [or ∞]	∞ [or 0]
ICA-w	Impute to create worst case scenario for experimental treatment	∞ [or 0]	0 [or ∞]
ICA-r	Impute incorporating available reasons for missing data	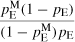	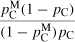

ICA – Imputed case analysis.

We first distinguish between two conceptual approaches to undertaking the imputations. The first is to impute an outcome for each missing participant, then to perform a standard analysis on the filled-out data set. The second approach is to impute risks of events for the groups of people with missing outcomes, and to calculate treatment effects from the observed and imputed risks. With small numbers of missing participants, the former strategy can be subject to large rounding error when assumptions about missing outcomes do not map directly onto individual participants, with unnecessary error in effect estimates. In fact, the standard methods for estimating treatment effects (e.g., OR, RR etc) allow nonwhole numbers of participants. For example, five missing participants might be divided into two and a half with the event, and two and half without the event. Following this strategy, the two conceptual approaches yield identical estimates of treatment effect. However, we will see in a later section that the two approaches lead to different standard errors, and hence different weights in the meta-analysis.

Two commonly used strategies are to assume that all missing participants experience the event, or that none of the missing participants experience the event [14]. These correspond to imputing risks of 1 and 0 for the missing participants, and we denote such imputed case analyses as ICA-1 and ICA-0, respectively (Table 2). Such assumptions may be appropriate when outcomes of missing participants can be predicted. For example, in trials of smoking cessation interventions it is common to assume that dropouts continue to smoke [15,16], although even here we would rarely believe the implicit assumption that all those who stop smoking provide outcome data.

An alternative imputation strategy is to assume that all missing participants have the same risk as the observed participants in the control group, a strategy we denote by ICA-*p*_C_ [13]. This may appear reasonable if none of the excluded participants had received the experimental treatment, or if those excluded from the experimental group stopped taking a treatment with reversible effects. However, similarity in received treatment is not a sufficient justification: missing and observed participants must also have no systematic differences in other characteristics that might be associated with the missing outcome values. This is a substantial assumption analogous to the missing at random assumption.

More rarely, one might assume that missing participants have the same risk as the observed participants in the experimental group, a strategy we denote by ICA-*p*_E_. This may be thought appropriate, for example, when participants from the control group are excluded because they started receiving the experimental treatment.

Imputing the same risks in both groups, using either the risk observed in the control group (ICA-*p*_C_) or those observed in the experimental group (ICA-*p*_E_), dilutes effect estimates, pulling them towards the null hypothesis. This can be seen by writing down the revised risks in the two groups, say 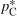 and 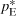. For example, imputing the control group risk throughout produces 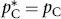 and (1)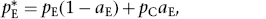 where *a*_E_ is the proportion of participants in the experimental group with missing data (see Table 1). Effect estimates are obtained by comparing the revised risks 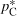 and 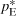. As an example, a revised estimate of the RR is (2)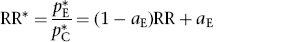 which is closer to 1 than RR, the RR among observed participants.

Rather than imputing according to a common risk across both groups, we could impute according to group-specific risks, a strategy we call ICA-*p*. Here missing participants from the control group are assumed to have the same risk as observed participants in the control group, and those missing from the experimental group to have the same risk as those observed in the experimental group. This is exactly the missing at random assumption and the effect estimate is the same as for available case analysis. However, the first conceptual approach, in which outcomes are imputed for individual missing participants, has the effect of scaling up the size of the data set and thus wrongly reducing standard errors, as discussed below.

#### Imputation according to available reasons for missingness

If reasons for participants being missing are available from each study, these may be exploited in an imputation scheme that combines aspects of the above five schemes. We propose a strategy, ICA-r, in which values missing due to different reasons may be imputed using different imputation strategies chosen from ICA-0, ICA-1, ICA-*p*_C_, ICA-*p*_E_ or ICA-*p*. For example, if it is known that patients have missing outcome assessments because they deteriorated to an extent necessitating their removal from the trial, they would be assigned to ICA-0 or ICA-1 (depending on the outcome definition) to reflect an unsuccessful outcome. On the other hand, patients who are missing for reasons unlikely to be related to treatment (for example, moving away from the area) might be assigned imputation scheme ICA-*p*. For participants whose data are missing due to death, we would make use of any available information on cause of death. Should this be absent, it may in some situations be reasonable to assume treatment failure, so that strategy ICA-0 or ICA-1 is employed. The method requires the subjective assignment of participants’ reasons for being missing to particular assumptions about their outcomes. We illustrate this strategy for the specific example of schizophrenia trials later.

It is straightforward to work out the imputed risk in each group when using reasons for missingness. If the proportions of missing participants, *a*_E_ and *a*_C_, are partitioned according to the reasons so that, for example, 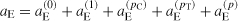 then the estimated risks among the missing participants are (3)
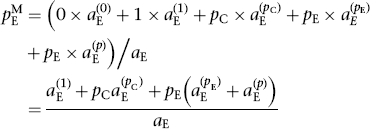
 in the experimental group and (4)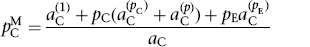 in the control group. Overall event rates in the two groups are then (5)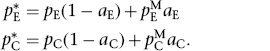 For studies that do not report reasons for missingness or cause of death, possible options include:

- determine the relative proportions of specific reasons for missingness across trials that do report them, and impute according to these proportions (this corresponds to calculating 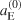, 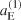, etc, across all studies providing reasons for missingness, and applying Equations (3) and (4) once to impute risks 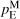 and 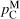 for use in the remaining studies);
- impute according to the relative proportions of specific reasons in the ‘most similar’ trial;
- impute according to the most common reason for missingness among all trials;
- use only an available case analysis.

In the example that follows, we follow the first of these strategies.

#### Best-case and worst-case scenarios

A common sensitivity analysis in the face of missing data is to impute outcomes to recreate the most extreme possible data sets, one reflecting the best-case scenario for the experimental treatment (ICA-b) and the other the worst-case scenario (ICA-w). The best-case scenario, for example, would assume missing participants in the experimental group had good outcomes and those in the control group had bad outcomes. Such imputation would provide the largest and smallest effect estimates compatible with the observed data.

As an aside, we remark that a rather different approach to dealing with missing data is to inflate standard errors of treatment effect estimates from available-case analyses. Gamble and Hollis have proposed an approach to meta-analysis based on the best-case and worst-case scenarios [17]. The most extreme lower and upper confidence interval limits from simple implementations of these two analyses are used to form an uncertainty interval for each study. These uncertainty intervals, treated as if they were confidence intervals, are converted into inflated standard errors, leading to reduced weights for use in the meta-analysis. The meta-analysis applies these weights to treatment effect estimates from an available case analysis. The reduced weights reflect the added uncertainty one might associate with data being missing.

### A generalization: the informative missingness odds ratio

We now provide a general framework for making assumptions about informatively missing data. This framework contains all the ICA methods so far considered as special cases (Table 2). The key idea is to specify the risk among the missing participants in the form of the odds ratio of the event among missing participants relative to the event among observed participants. This is allowed to be different in the two groups. We refer to these as informative missingness odds ratio (IMOR), and denote them IMOR_E_ and IMOR_C_ for the two treatment groups.

This approach provides a generalization of the above imputation strategies; connections with the specific methods provided in Table 2. Our formulation differs from a similar one by Magder [18]. His ‘response probability ratio’ is the ratio of the probabilities of nonmissingness between those with events and those without events, whereas our IMOR is an odds ratio. The odds ratio has the advantage that it cannot predict probabilities less than zero or more than one [9]. Generalizations of our approach have been studied extensively in the statistical literature. Rotnitzky *et al*. considered regression modeling of longitudinal data using a sensitivity analysis over parameters that govern the degree of informative missingness [19]. Reduced to the setting of a single binary outcome and a single binary covariate (e.g., randomized group), their sensitivity parameters correspond to our IMORs. The inherent nonidentifiability of missing data problems has been stressed by Little [20]. A natural extension is to quantify prior uncertainty about IMORs [8,21–24].

### Weighting schemes for imputed case analyses

We now outline how standard errors can be obtained. We consider first the approach in which outcomes are filled in for the missing participants. We observe this to be in common use in the field of meta-analysis, so some discussion is relevant. The simplest approach is to treat imputed data as if they were known, and calculate standard errors for the studies in a meta-analysis in the usual way. We shall refer to this approach as scheme W1:

W1. *Naïve approach*: Treat the imputed case data set as if it was completely observed so that uncertainty associated with imputing missing values is ignored.

This scheme is inappropriate since it fails to recognize that some data are observations and others are imputed. This is particularly clear if we compare ICA-*p* with ACA: the imputed data do not change the effect estimate and serve only to inflate the sample size and reduce standard errors. Indeed, in most cases the imputation strategies we have outlined reduce standard errors using this scheme; an exception is ICA-0 in conjunction with risk ratios. We consider two simple alternative schemes as follows.

W2. *A hybrid approach*: Here we aim to use standard errors corresponding to the amount of observed data. We determine effect estimates from the imputed case data set, but use standard errors directly from the available case analysis.

A disadvantage of W2 is that the imputations may alter risks, and these risks should be reflected in the standard errors. We therefore consider:

W3. *A data-set re-sizing approach*: We determine risks (for experimental and control) from the imputed case data set, but apply these to the numbers of participants whose outcome is known (i.e., from the ACA data set) to create a revised 2 × 2 table. This re-sized data set then forms the basis for the application of standard methods. The row totals in the revised 2 × 2 table will be identical to those in the available case analysis.

Turning now to the alternative conceptual approach to the imputation, we consider theoretical approximate standard errors, derived conditional on the IMORs. The risks rather than the outcomes are now imputed, and these may be derived from one of the ICA approaches listed in Table 2, or from IMORs external to the study.

W4. *IMOR-based approach*: Standard errors are based on application of IMORs to observed risks, taking into account uncertainty in observed risks and missingness probabilities.

We derive standard errors for this approach in the Appendix. For imputation with fixed IMORs (such as ICA-0, ICA-1, ICA-*p*, ICA-b and ICA-w), this approach yields statistically correct standard errors. For imputations ICA-*p*_C_, ICA-*p*_E_, and ICA-r, the standard errors are conditional on the IMORs and ignore the fact that the IMORs are computed from the data. Instead, standard errors can be calculated for ICA-*p*_C_, ICA-*p*_E_ directly from Equations (1) and (2), and related methods could be used for ICA-r. However, the resultant standard errors are often smaller than if complete data were observed. This is a logical but unsatisfactory consequence of the assumption that the unobserved outcomes have exactly the same expectation in both groups. Much more plausible results are obtained by conditioning on the IMORs.

Weights awarded to studies in a meta-analysis may be derived from the standard errors. In a common-effect meta-analysis, where a common (fixed) effect is assumed, the weights are typically calculated as inverse squares of the standard errors. Random-effects meta-analyses are more difficult to predict since changes in effect estimates may result in changes in the extent of heterogeneity among studies, which is incorporated into the study weights.

## Application to haloperidol trials

We apply the above methods to a meta-analysis of RCTs comparing haloperidol with placebo in the treatment of schizophrenia. The antipsychotic properties of haloperidol were discovered in the 1950s and the drug was believed to be effective and well tolerated compared with alternatives. However, trials involving schizophrenic patients are prone to high proportions of missing data due, among other reasons, to poor compliance of patients, rigid implementation of RCT protocols and side effects. A Cochrane review of haloperidol forms the basis of our data [25].

### Meta-analysis methods

We used only trials identified by the Cochrane review, which sought controlled trials in patients with schizophrenia or similar serious psychotic illnesses randomized to any dose of haloperidol or placebo. A comprehensive search strategy included multiple electronic databases, cited reference searching, hand searching of journals and direct contact with investigators. Twenty trials were included in the review (at the end of 2002). The original review excluded trials with greater than 50% missing data, and we maintained this exclusion because suitable clinical outcomes were not reported. We retrieved the main publication of each of the 20 included trials and two of us (JH and AW) independently extracted information on: ran-domized sample size; a dichotomous clinical outcome of global improvement (choosing the primary outcome of the study when specified); numbers of missing data; and reasons for missing data. Discrepancies were resolved by discussion, with arbitration by IW when appropriate. Some arbitrary decisions were necessary. We defined clinical improvement as ‘moderate’ (or ‘good’) to ‘marked’ (or ‘excellent’) [26,27]; we ignored the cross-over design of one trial [26] and assumed in one study that ambiguous percentages reflected ratios of patient numbers [28]. Since our aim is to evaluate effects of missing data on clinical outcomes, we discarded trials from which we could not obtain dichotomous data on clinical improvement.

We focus on risk ratios for clinical improvement, as used in the original Cochrane review, so that risk ratios greater than 1 reflect a beneficial effect of haloperidol. Our meta-analyses are simple weighted averages of log RR estimates. We mainly present results for analyses assuming a common effect (so-called ‘fixed-effect’ meta-analyses) since the implications of the missing data are easier to interpret. When we refer to random-effects meta-analyses, these incorporate method of moment estimates of among-study variance [29]. We note without further comment that potentially important heterogeneity of effects is present in the data set, along with an apparent relationship between effect size and study size. Prior to all analyses we applied a continuity correction to trials in which r_E_, f_E_, r_C_ or f_C_ (Table 1) were zero, adding a half to each of these values.

Our analysis using reasons for missingness (ICA-r) assigned reported reasons to imputation strategies as described in Table 3. In some trials the reasons for missingness were available for precisely the missing participants, and the imputation was then straightforward. In other trials, the reasons for missingness were given for a different subset of participants, for example when clinical outcome and dropout were reported for different time points. In such cases we applied the proportion in each classification to the missing population in that trial. In trials that did not report any reasons for missingness, the overall proportion of reasons from all other trials was used.

**Table 3:** Assignments of reasons for missingness to different imputation strategies for analysis of the haloperidol data

Classification of reasons	Imputation strategy
Lack of therapeutic benefit, lack of efficacy, relapse, insufficient/inadequate response, behavioral deterioration.	ICA-0
Positive response.	ICA-1
Adverse experience, refusal, withdrawal of consent, protocol violation, patient ran away, patient uncooperative, patient decision, skin rash, tuberculosis, side effects, noncompliance.	ICA-p_C_
Loss to follow-up, administrative reasons, failure to report to hospital, patient sleeping, other.	ICA-p

ICA – Imputed case analysis.

## Results

We were left with 17 trials providing data on clinical improvement, and basic results from these are listed in Table 4 [26–28,30–43]. Note that only two trials have substantial amounts of missing data. The study by Beasley reported outcomes for only 81 (59%) out of 137 participants [31]. The study by Selman *et al*. reported outcomes for only 29 (50%) out of 58 participants [40]. These two studies were among four that specifically stated they involved acutely ill participants, but were otherwise not markedly different in characteristics from the other studies.

**Table 4:** Data from 17 trials of haloperidol for schizophrenia

Trial	Haloperidol				Placebo			
	Improved	Not improved	Missing	Total	Improved	Not improved	Missing	Total
	r_E_	f_E_	m_E_	N_E_	r_C_	f_C_	m_C_	N_C_
Arvanitis [30]	25	25	2	52	18	33	0	51
Beasley [31]	29	18	22	69	20	14	34	68
Bechelli [27]	12	17	1	30	2	28	1	31
Borison [32]	3	9	0	12	0	12	0	12
Chouinard [33]	10	11	0	21	3	19	0	22
Durost [28]	11	8	0	19	1	14	0	15
Garry [34]	7	18	1	26	4	21	1	26
Howard [35]	8	9	0	17	3	10	0	13
Marder [36]	19	45	2	66	14	50	2	66
Nishikawa 82 [37]	1	9	0	10	0	10	0	10
Nishikawa 84 [38]	11	23	3	37	0	13	0	13
Reschke [39]	20	9	0	29	2	9	0	11
Selman [40]	17	1	11	29	7	4	18	29
Serafetinides [41]	4	10	0	14	0	13	1	14
Simpson [42]	2	14	0	16	0	7	1	8
Spencer [43]	11	1	0	12	1	11	0	12
Vichaiya [26]	9	20	1	30	0	29	1	30

**Figure 2:**
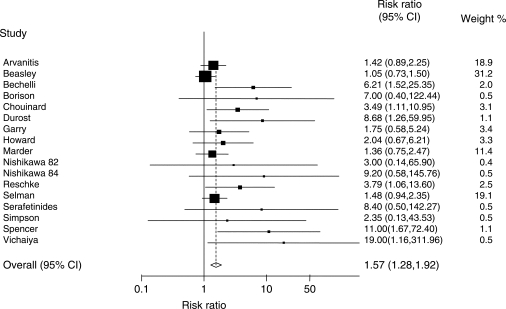
Meta-analysis (assuming a common effect) of available case analyses (ACA) from each of the haloperidol trials

An ACA assuming a common RR across studies yields a meta-analytic estimate of 1.57 (95% confidence interval from 1.28 to 1.92), indicating strong evidence of a clinical improvement due to haloperidol (Figure 2). Table 5 shows results of meta-analyses using the various imputation strategies and weighting schemes described above. The overall conclusion is robust to most strategies, the extreme worst-case scenario being the only analysis with a 95% confidence interval including a RR of 1. However, the point estimates are variable. For example, imputing only failures for missing outcomes (ICA-0) increases the RR estimate to approximately 1.90, and imputing only successes (ICA-1) decreases it to between 1.16 and 1.41, depending on the choice of weighting scheme.

**Table 5:** Comparison of different imputation and variance inflation strategies in the haloperidol meta-analysis: point estimates of RR for clinical improvement in Beasley and Selman trials with percentage weights awarded to them in the meta-analysis; and estimated common risk (Pooled RR) with 95% confidence interval

	Beasley					Selman					
	RR	Weight (%)				RR	Weight (%)				Pooled RR (95% CI)			
		W1	W2	W3	W4		W1	W2	W3	W4	W1	W2	W3	W4
ACA	1.05	31.2				1.48	19.1				1.57 (1.28, 1.92)			
ICA-0	1.43	25.0	31.2	17.0	25.0	2.43	10.4	19.1	5.2	10.4	1.90 (1.51, 2.39)	1.88 (1.54, 2.30)	1.94 (1.50, 2.50)	1.90 (1.51, 2.39)
ICA-1	0.93	35.8	31.2	37.1	35.8	1.12	47.4	19.1	34.0	47.4	1.16 (1.04, 1.29)	1.41 (1.15, 1.72)	1.24 (1.07, 1.44)	1.16 (1.04, 1.29)
ICA-p_C_	1.03	37.5	31.2	31.8	32.6	1.30	27.4	19.1	17.2	14.9	1.40 (1.18, 1.65)	1.51 (1.24, 1.85)	1.52 (1.24, 1.87)	1.53 (1.24, 1.88)
ICA-p_E_	1.02	25.3	31.2	24.8	19.7	1.14	51.4	19.1	36.4	50.1	1.27 (1.11, 1.46)	1.46 (1.20, 1.79)	1.40 (1.17, 1.67)	1.33 (1.14, 1.56)
ICA-p	1.05	35.6	31.2	31.2	31.2	1.48	31.6	19.1	19.1	19.1	1.46 (1.24, 1.72)	1.57 (1.28, 1.92)	1.57 (1.28, 1.92)	1.57 (1.28, 1.92)
ICA-r	1.35	28.4	31.2	21.4	27.1	1.77	21.9	19.1	12.1	16.4	1.76 (1.44, 2.15)	1.75 (1.43, 2.14)	1.84 (1.46, 2.33)	1.79 (1.44, 2.21)
ICA-b	2.51	30.1	31.2	20.0	30.1	4.00	11.1	19.1	5.4	11.1	2.42 (1.95, 3.00)	2.56 (2.09, 3.13)	2.30 (1.80, 2.94)	2.42 (1.95, 3.00)
ICA-w	0.53	33.3	31.2	28.4	33.3	0.68	26.6	19.1	19.5	26.6	0.94 (0.79, 1.12)	1.04 (0.85, 1.27)	1.08 (0.89, 1.32)	0.94 (0.79, 1.12)
IMOR_E_ = 2,	1.00	36.8	31.2	34.3	35.2	1.32	36.8	19.1	23.6	26.2	1.34 (1.16, 1.55)	1.51 (1.24, 1.85)	1.45 (1.20, 1.74)	1.42 (1.19, 1.69)
IMOR_C_ = 2														
IMOR_E_ = ½,	1.12	33.3	31.2	27.3	27.5	1.74	25.9	19.1	14.7	14.1	1.61 (1.34, 1.93)	1.65 (1.35, 2.01)	1.70 (1.37, 2.12)	1.70 (1.37, 2.12)
IMOR_C_ = ½														
Gamble-Hollis	1.05	6.6				1.48	4.4				2.02 (1.51, 2.70)			

ACA – available case analysis; ICA – Imputed case analysis; W1 – W4 as defined in the text. In trials with no successes or failures, 0.5 has been added to each of r_E_, f_E_, r_C_, and f_C_ (so that N_E_ and N_C_ increase by 1).

Some implications of the various weighting schemes for the two studies with the most missing data [31,40] are also provided in Table 5. These two studies drive the differences among the various strategies and weighting schemes. In the ACA they contribute just over 50% of the weight between them. Using naïve weights (W1) with the various imputation strategies illustrates that the implications of this weighting scheme depend on the imputation method. Weights for these two studies increase when successes are imputed and decrease when failures are imputed (and weights for the studies with few or no missing data change in the opposite direction). The effects on weights when both successes and failures are imputed depend on the existing and imputed risks and are less easy to predict. Weighting scheme W2 simply uses the same weights as the ACA. Weighting scheme W3 generally, though not always, gives the two studies less weight than W1, but in common with W1 it reflects the risks obtained after imputing missing outcomes. The final weighting scheme, W4, theoretically derived, is similar to W1 when IMORs are extreme, so that risks of 0 or 1 are imputed (ICA-0, ICA-1, ICA-b, ICA-w). For other analyses, W4 appropriately down-weights studies with missing data compared with the naïve scheme W1.

We repeated the analyses using a random-effects meta-analysis model (not shown). We observed similar patterns, but make two remarks. First, all point estimates were larger due to a tendency for larger risk ratios to be found in smaller trials (which are awarded relatively more weight in a random-effects meta-analysis). Second, there was less variation in findings across the various imputation strategies and weighting schemes. This might be expected given that the studies with substantial amounts of missing data had large weights in the common-effect meta-analysis and hence receive smaller weights in a random-effects meta-analysis. The effect of different imputation strategies will also differ between common-effect and random-effects meta-analyses when the strategies introduce or remove substantial heterogeneity. Heterogeneity is affected by the sizes of both point estimates and their standard errors. In the haloperidol example there was little variation in heterogeneity (data not shown), other than on implementation of the worst-case scenario, ICA-w (when standard χ^2^ statistics were approximately tripled).

Table 5 also provides some results for specific values of (IMOR_E_, IMOR_C_), assuming values of (2, 2) and (½,½). These assume the odds of clinical improvement among missing patients is either double or half the odds of clinical improvement for observed patients. The results tend in the expected directions. For example, with IMORs below 1 in both groups the results are intermediate between ACA and ICA-0. Note that assuming IMORs of (1, 1) is equivalent to the ICA-*p* analysis, and the ICA-*p* analysis is itself equivalent to the ACA analysis under weighting scheme W2.

The sensitivity analysis of Gamble and Hollis (Table 5) gives a pooled RR of 2.02, which is somewhat larger than most of the imputed case analyses. This is because the method gives considerably smaller weights to the Beasley (6.6%) and Selman (4.4%) studies which both have RR estimates on the low side.

Many of the assessments of global improvement in these trials were derived by dichotomizing scales. Four of the studies [28,30,33] reported having imputed scores on these scales by carrying forward the last available observation. We were unable to account for this in our analysis of the published results, so the true amount of missing data is greater than we have assumed. Furthermore, since repeated measurements were made in many of the trials, valuable information about participants missing from the primary timepoint of interest would in theory be available from earlier time points. Again, the summary binary data available to us did not permit exploitation of this.

## Recommendations

We have described and implemented a number of strategies for addressing missing binary outcome data from clinical trials in a meta-analysis. Here we discuss the relative merits of the methods and make suggestions for practice, both for primary meta-analysis and for sensitivity analyses. We formulated some principles to guide our selection:

Precision. The standard error for an effect estimate from a particular study after accounting for missing data should not be smaller than in the ACA, and ideally should be larger. We suggest this principle to ensure that appropriate uncertainty induced by missing data is carried forward into the meta-analysis. This rules out weighting scheme W1, which we believe to be commonly implemented, as it treats imputed data as known and increases precision of effect estimates. (Note that assuming missing values to be successes, as in ICA-1, should logically reduce standard errors, but because one can never be sure of such an assumption, we propose not accepting the standard error reduction.)

Reducing bias by making use of relevant information. In some cases the bias (or at least its direction) in ACA can be anticipated. External information relevant to the likely bias should be used when available. Such information might include reasons for missingness, evidence from related studies or subject expertise.

Scale independence. A good strategy should be applicable whether the meta-analysis is conducted on the RD, RR or OR scale.

Simplicity. A strategy should be simple to understand and straightforward to implement, for example by being possible in widely used software.

### Proposal for a principal meta-analysis

We believe that an ACA should generally be presented as a point of reference. Sometimes this will be considered suitable for a primary analysis. However, we suggest as a preferable primary analysis one that emphasizes the second of our principles. Thus we suggest the strategy of imputing missing data according to reasons for missingness (ICA-r). The categorization of reasons should ideally be specified in advance of seeing the data. Although, this involves subjective judgments about the true outcomes of missing participants, we feel that this approach may most closely represent what would have been observed. Furthermore, if there is a diverse mixture of reasons for missingness, as was the case in our example, then the approach essentially averages over several of the specific imputation strategies. We propose using weighting scheme W4, in which the uncertainties in the observed risks and the extent and assumptions of missingness are incorporated into the analysis. Our primary analyses are presented in Table 6.

**Table 6:** Proposed analysis strategy with missing data: Results of meta-analyses assuming common RR applied to 17 haloperidol trials. Meta-analyses produce estimates of RR for clinical improvement with 95% confidence interval. Inconsistency of risk ratios across studies is measured using I^2^ [45]. Results for Beasley and Selman trials are estimates of RR and percentage weights awarded to them in the meta-analysis

		Beasley		Selman		Meta-analysis	
		RR	Weight (%)	RR	Weight (%)	Pooled RR (95% CI)	I^2^ (95% CI)
Reference analysis	ACA	1.05	31.2	1.48	19.1	1.57 (1.28, 1.92)	41 (0, 66)
Proposed analysis (weight W4)	ICA-r	1.35	27.1	1.77	16.4	1.79 (1.44, 2.21)	27 (0, 59)
Sensitivity analysis	IMOR_E_ = 2, IMOR_C_ = 2	1.00	31.2	1.32	19.1	1.51 (1.24, 1.85)	44 (0, 67)
(weight W2)	IMOR_E_ = ½, IMOR_C_ = ½	1.12	31.2	1.74	19.1	1.65 (1.35, 2.01)	38 (0, 64)
	IMOR_E_ = ½, IMOR_C_ = 2	0.85	31.2	1.28	19.1	1.41 (1.15, 1.73)	52 (1, 71)
	IMOR_E_ = 2, IMOR_C_ = ½	1.32	31.2	1.80	19.1	1.76 (1.44, 2.16)	29 (0, 60)

ACA – available case analysis; ICA – Imputed case analysis; IMOR – informative missingness odds ratio for experimental group (E) or control group (C).

An alternative approach, in a similar vein, would be to express explicitly the uncertainty about IMORs by making use of prior distributions in a Bayesian analysis [24]. Prior distributions may be available from external evidence sources, from heuristic arguments, or may constitute prior distributions formally elicited from subject experts. The determination of the prior distribution would ideally involve consideration of the methodology, proportions of missing data and any available reasons for missingness from each component study. The IMORs may be the same or different for different studies. Note that this approach can incorporate judgments that, for example, all missing outcomes should be assigned a value of 1 (IMOR = ∞) or a value of 0 (IMOR = 0), as in the example of smoking cessation studies we cited earlier.

## Proposals for simple sensitivity analysis

Analyses that attempt to account for unobserved data should be assessed in sensitivity analyses. A thorough sensitivity analysis should separate two dimensions: (1) the effect of allowing for missing data on the effect estimates from the individual studies; and (2) the effect of allowing for missing data on the standard errors (and hence weights) of these estimates. This is because the result of a meta-analysis is affected jointly by the magnitude of effect estimates from individual studies and by their standard errors. Here we discuss a simple strategy that might be adopted in practice.

**Figure 3:**
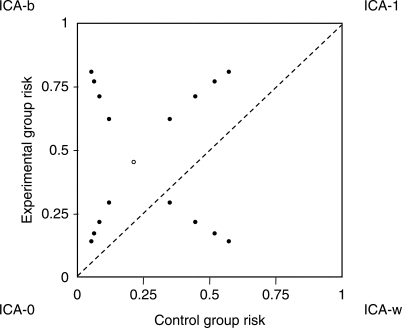
L’Abbé plot providing graphical representation of the proposed sensitivity analysis strategy, representing risks to be applied to missing participants. The dotted line represents absence of a treatment effect. The open circle corresponds to a experimental group risk of 0.46, and a control group risk of 0.21, reflecting the overall risks among the haloperidol trials. Filled circles represent combinations of IMORs of 2,1/2 (nearest to the open circle); 3,1/3; 4,1/4; and 5;1/5 (nearest to the corner). In this example, points above the dotted line represent superiority of haloperidol, and points below represent superiority of placebo. Note that choosing larger IMORs (with their reciprocals) leads to traveling along curved paths towards the corners. The corners reflect four of the imputation strategies described earlier, where IMORs are combinations of 0 and ∞

The four extreme imputation approaches of assuming that all missing participants had an event (ICA-1) or no event (ICA-0) and the best-case (ICA-b) and worst-case (ICA-w) scenarios provide limits on effect estimates compatible with the data. We consider these to be rather unlikely scenarios, especially when there are many missing participants. Instead, we suggest selecting IMORs for the two groups that cover realistic situations. Our strategy is illustrated in Figure 3, following the graphical sensitivity analysis proposed by Hollis [44]. This is a L’Abbé plot (experimental group risk vs. control group risk) for risks to be applied to the missing participants. The corners of the plot represent the extreme imputation strategies, and all points on the plot correspond to pairs of IMOR_E_ and IMOR_C_. For a given ‘starting point’, corresponding to the primary analysis (the open circle, typically the ACA), we move in four directions towards the corners of the plot. Moving towards the ICA-0 and ICA-1 corners involves assuming the same IMORs in the two treatment groups; moving towards the best-case and worst-case corners involves assuming different IMORs in the two treatment groups. We achieved these directions by taking IMOR_C_ = IMOR_E_ or IMOR_C_ = 1/IMOR_E_, respectively. We propose using weighting scheme W2 (ACA weights) so that only the point estimates are affected by the different IMORs. A selection of combinations of IMOR_E_ and IMOR_C_ should be used, based on the views of experts in the field. In Table 6 we illustrate IMOR combinations involving 2 and ½ for the haloperidol data, corresponding to the four innermost filled circles in Figure 3. This demonstrates that the results of the ACA are robust to 2-fold differences in risks between outcomes among the missing participants and outcomes of observed participants.

To evaluate the effects of missing participants on the weights awarded to the studies, the inflated confidence intervals of Gamble and Hollis might be used. This can lead to excessively wide confidence intervals and considerable down-weighting of studies with missing data. We also propose a more statistical treatment of the problem, providing a comprehensive sensitivity analysis strategy using correlated prior distributions for the two IMORs [24].

## Discussion

We have overviewed methods for dealing with missing dichotomous outcome data in meta-analysis of clinical trials, and illustrated a strategy for primary analysis and a series of sensitivity analyses. We make extensive use of the notion of an informative missingness odds ratio, describing the relative risks among missing and observed participants. A wide variety of imputation schemes are seen to be special cases of this general approach. The methods we present can be programmed into basic statistical software or a simple spreadsheet.

In applying different strategies to an example of clinical trials of haloperidol for schizophrenia we have established that the results of this particular meta-analysis are robust to reasonable assumptions concerning the outcomes of missing participants. The data set is typical of many meta-analytic data sets, since it contains studies of various sizes and variety in the degree of missingness across studies. However, there exist many meta-analyses with more severe rates of missingness, and with a larger proportion of studies having high rates of missing data. It is likely that in some cases the substantive results would be changed by sensitivity analysis. Using all available reasons for missingness, a strategy rarely used in meta-analysis, may help to make optimal use of such data sets.

The ideas could be extended to analysis of other types of data. For example, for continuous outcomes, ‘informative missingness differences in means’ or ‘informative missingness ratios of means’ might replace the IMORs. These could be used to impute missing outcomes that are similar, bigger or smaller than the observed outcomes within any particular treatment group. The best-case and worst-case scenarios may however not be possible unless there are known limits to the outcome (as in psychometric scales, for example).

In conclusion, we propose a systematic approach to dealing with missing outcome data in meta-analysis. Adoption of these strategies should allow a transparently obtained ‘best-guess’ primary analysis, and a series of sensitivity analyses to evaluate the robustness of the conclusion to how the missing data were handled.

The procedures described are easily implemented using the Stata programme metamiss.ado which is available from http://www.mrc-bsu.cam.ac.uk/BSUsite/software/.

## Appendix: Standard errors of treatment effect estimates based on observed risks and IMORs

We denote overall risks in the experimental and control groups by  and , respectively. These risks result from combinations of observed event rates and imputation assumptions for missing participants. The treatment effects, *RD**, *RR**, and *OR**, are estimated directly from  and . To estimate their variances we use standard Taylor series approximations, taking the covariance between  and  to be zero, corresponding to independent IMORs for the experimental and control groups.

The task is to obtain estimates of  and .

IMOR_E_ is defined as the odds of outcome in the missing individuals divided by the odds of outcome in the observed individuals in the experimental group. For a given IMOR_E_, we can estimate the risk in the missing individuals in the experimental group as

so our estimate of the true risk, analogous to (1) or (5), is

(6)

We can obtain the variance of the true risk as

which is based on a further Taylor approximation to (6): the terms in large parentheses are the partial derivatives of  with respect to *p*_E_ and *a*_E_, respectively, and the terms outside them are variances of *p*_E_ and *a*_E_, respectively. A similar calculation gives .

A generalization allows for different IMORs in different individuals, to cover different reasons for missingness. Let _Er_ be the proportion of all individuals in the experimental group who are missing for reason r (so that ). Let IMOR_Er_ be the IMOR specific to these individuals, then their risk is

and the estimated true risk among all individuals in the experimental group is

The variances are given by

and similarly for the control group. These formula simplify to the previous formulae when there is a single reason.

To deal with trials in which the number of successes or the number of failures is zero in one group, a continuity correction (adding 0.5) may be applied to numbers of successes and failures either before or after calculating the imputed risks. In our analyses we have made the correction before imputing missing outcomes, but only to 2 × 2 tables that would have had a zero cell after imputing any successes or failures using ICA-0, ICA-1, ICA-b or ICA-w.
